# The early loading of different surface-modified implants: a randomized clinical trial

**DOI:** 10.1186/s12903-021-01498-z

**Published:** 2021-04-26

**Authors:** Kinga Körmöczi, György Komlós, Petra Papócsi, Ferenc Horváth, Árpád Joób-Fancsaly

**Affiliations:** 1 Oral and Maxillofacial Department, Faculty of Dentistry, Semmelweis University, Mária str 52, 1085 Budapest, Hungary; 2 Department of Public Health, Faculty of Medicine, Semmelweis University, Nagyvárad Sqr. 4. 13th Floor, 1089 Budapest, Hungary

**Keywords:** Early loading, Surface modification, Sand-blasted/acid-etched surface, Hydroxyapatite surface

## Abstract

**Background:**

Various surface treatment options have been adopted with the aim to improve osseointegration, reducing the overall treatment time. Implant stability of early loaded implants with different modified surfaces was compared in the present study.

**Methods:**

Patients were selected from the Department of Oro-Maxillofacial Surgery and Stomatology at Semmelweis University. Patients randomly received SA (alumina sandblasted and acid-etched), NH (bioabsorbable apatite nanocoating) or SLA (large-grit sandblasted and acid-etched) surface implants. Outcome measures were: implant success, implant stability, and periodontal parameters. The implant stability was measured at the time of implant placement (primary stability) and six weeks after (prothesis delivery, secondary stability). Osstell and Periotest were applied to take all the measurements. The primary and secondary stability were compared in the three study groups Finally the periimplant probing depth appearing after three months of loading was checked on 6 points around to the implant-supported prostheses. Shapiro–Wilk and Mann–Whitney tests were used for the comparison between the study groups.

**Results:**

A total of 75 implants with different length and diameter were inserted into various positions. One implant failed spontaneously at the fourth week after implant placement. The survival rate was 98,7%. Comparing the primary and secondary stability values, the data were significantly improved in every groups. The difference was the highest in the NH group, however, this difference was not significant compared to the two other groups. Good periodontal parameters were experienced in all the tested implants, independently by the groups.

**Conclusions:**

With the limitation of the present study, all the implants showed improved stability six weeks after implant placement. A trend of higher result was found for the NH group. Further studies with longer follow-up are needed to confirm this preliminary results.

*Trial registration*: Current Controlled Trials ISRCTN13181677; the date of registration: 04/03/2021. Retrospectively registered.

## Background

The direct integration of titanium implants into the bone was first reported by Rudy et al. [20]. After various animal experiments, dental implants began to be used in everyday dental practice from 1965 [4]. Osseointegration was firstly described in 1976 as the direct contact between machined surface implant and the surrounding bone at microscopy level. The observation of a direct bone-implant contact (BIC), confirmed by Albrektsson et al. with electron microscopy, was the most important discover in dental implantology [1]. Machined surface implants were characterized by low BIC therefore, implant surface modifications were suggested to enhance osseointegration, particularly in challenging clinical scenarios. The ideal form, utilized material, the biological indicator function of dental implant surfaces is a main focus of basic research in implant dentistry [5, 7, 8, 12].

Dental implant surfaces can be defined by their roughness (Sa), chemical composition, and by the physical and mechanical implant features. Surface roughness of implants can be categorized into three basic levels: minimally rough: 0.5–1 μm, moderately rough: 1–2 μm, rough: greater than 2 μm [2]. Moderately rough surface, compared to the previously used rough and smooth surfaces, has shown better clinical results by improving osseointegration, reducing the early failure rates [10, 26]. The combined large-grit sand-blasted acid-etched procedure is still one of the most frequently used surfaces modifications, which creates micro-sized surface elements of about 1.5 μm Ra value, showing a positive influence on osseointegration in several studies [14, 16, 17]. The effect of micrometer-sized structures on cells has been clarified, and the protein layer formation ability of nanometer-sized structures has been proven in animal experiments, although the latter mechanism is still unknown [12, 13, 22, 25]. Moreover, there remain some unexplained questions related to the clinical effects of current surface structures on the surrounding bone [11, 15, 27]. The osseointegration-stimulating effect of biological surface modifications (e.g., tricalcium phosphate and hydroxyapatite) has been frequently reported, however, the application of nanometer-sized biomaterials onto the implant surface is still under investigation [18, 19, 21].

The purpose to combine SLA surface with the osteoinductivity-features of hydroxyapatite (HA) is to take advantages of both processes. The hypothesis was that a nano coating of HA turns the bioinert titanium implant to be more hydrophilic (bioactive), making the implant able to overcome unfavorable conditions during undisturbed healing or earlier loading [23]. Previous investigations reported some failures using HA modified surface, nevertheless, the bonding strength was too low (20–35 MPa) resulting in the HA detachment. The novel HA coating was developed to be resorbable enhancing osseointegration process with no improved risk of implant failure. In vivo preliminary researches showed encouraging short and long-term results [28].

The ultimate goal of surface development is to achieve the quick, safe, and long-term formation of the secondary stability allowing for early prosthetic loading [25]. Many investigations and systematic reviews found that early loading did not negatively influence the osseointegration. Soft and hard tissues were reported very stable around the early loaded implants, similarly to conventional loading [9].

The aim of this randomized controlled trial was to investigate whether the implant surface characteristics influence the osseointegration process, up to six weeks after dental implant insertion.

## Methods

### Study design

A randomized prospective clinical trial was conducted at the Department of Oro-Maxillofacial Surgery and Stomatology at Semmelweis University to evaluate the effects of the SA (alumina sandblasted and acid-etched, Osstem Implants, Seoul, South Korea), NH (bioabsorbable apatite nanocoating, Osstem Implants) or SLA (sandblasted, large-grit and acid-etched, Straumann, Basilea, Swiss) surface on secondary implant stability of early loaded implants. Patients with good general health aged 18 or older with at least one missing tooth and requiring an implant supported fixed partial denture were included in the study. Written informed consents were signed by all participants. The study was approved by National Institute of Pharmacy and Nutrition (reference number: OGYÉI/55,197/2019), all treatments were performed according to Helsinki declaration (2013).

Inclusion criteria were: patients with an edentulous site requiring implant supported fixed partial dentures, good patient’s compliance, good or moderate oral hygiene, complete mucosal healing at the study site, previous tooth extraction performed between two months and one year prior to implant placement. Exclusion criteria were: general contraindications to oral surgery (i.e. non-controlled systematic diseases, chemo- and radiotherapy in the head and neck region, previous and ongoing bisphosphonate or denosumab therapy), incomplete mucosal healing at the study site, need for bone grafting, previous socket preservation or ridge augmentation, > 5 mm of periodontal pockets at adjacent teeth, poor oral hygiene, smoking more than 10 cigarettes per day.

### Clinical protocol

The medical and dental history of patients was registered. Before implant placement, all the necessary dental treatments were completed. The informed consent was fulfilled and signed by each patient. To evaluate the quantity and quality of the bone a preoperative low-dosage Cone Beam Computer Tomography (CBCT) scan was taken. All the surgical procedures were performed by three calibrated and experienced surgeons (ÁJF, GYK, KK). Implants were placed under local anaesthesia (4 ml Ultracain DS Forte, Sanofi Aventis, Paris, France). Then, a midcrestal incision was made continued in the intracrevicular sulcus of the adjacent teeth. In the first group (SA group) SA implants (Osstem implant were inserted. This implant is featured with a surface sand blasted with aluminium oxide grains of 250–500 μm and etched with hydrochloric acid or sulphuric acid. The Ra value is 2.5–3.0 μm. In the second group (NH group) NH implants (Osstem implant) were inserted. The characteristic of this hydrophilic surface is that the sand-blasted surface is covered with a 10 nm thick hydroxyapatite layer, which has the ability of bio absorption in the human body. The bonding strength of the HA layer is 75 MPa. The Ra value is 2.5–3.0 μm. In the third group (SLA group) SLA implants (Straumann implant) were inserted. The Ra value is 1.42 μm. Implant osteotomy was performed according to the manufacturers’ protocols. Implants of different lengths and diameters were chosen according to patients’ individual needs. All the implants were inserted using a manual wrench torque and the healing abutments were inserted. Wound closure was performed by simple interrupted suture. A postoperative periapical radiograph was taken. Fourteen days after surgery, sutures were removed. Six weeks after implant placement the patients were controlled and the secondary stability was measured. Three days after a definitive screw-retained fixed partial denture was delivered. Three months after definitive prothesis delivery clinical parameters were recorded. After study completion, patients were enrolled to a lifetime supportive therapy with visits every four to six months.

### Outcome measures

**Fig. 1:**
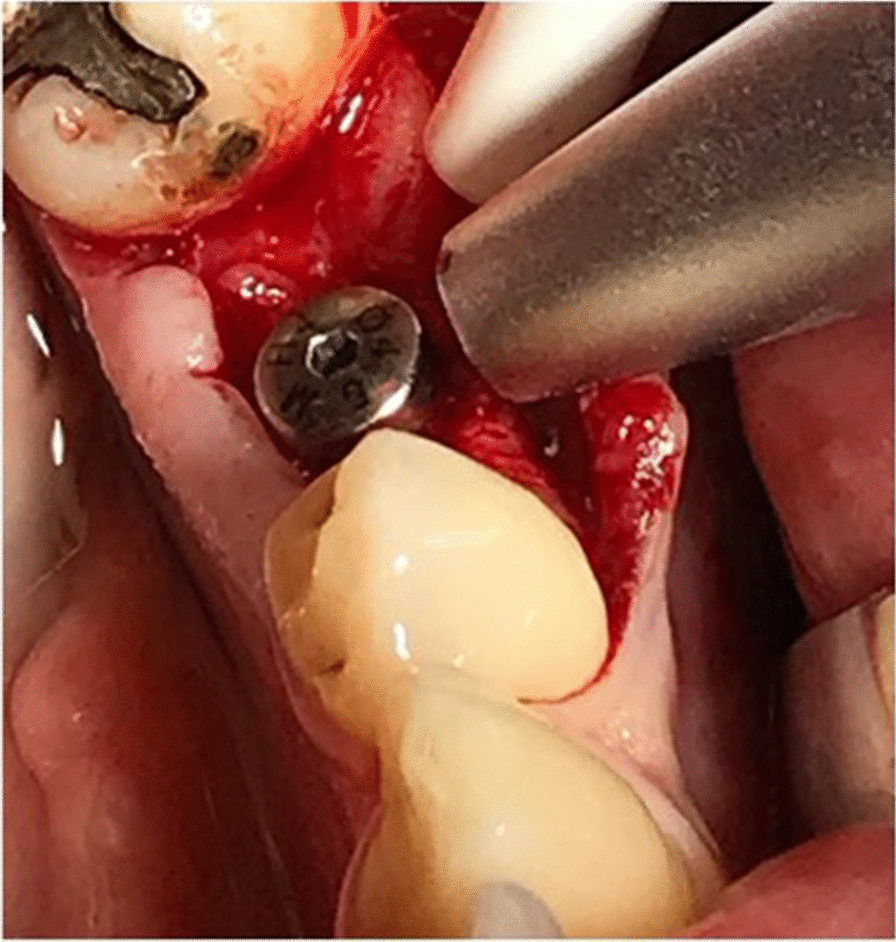
Measuring of the primary stability immediately after implant insertion with the Periotest

**Fig. 2:**
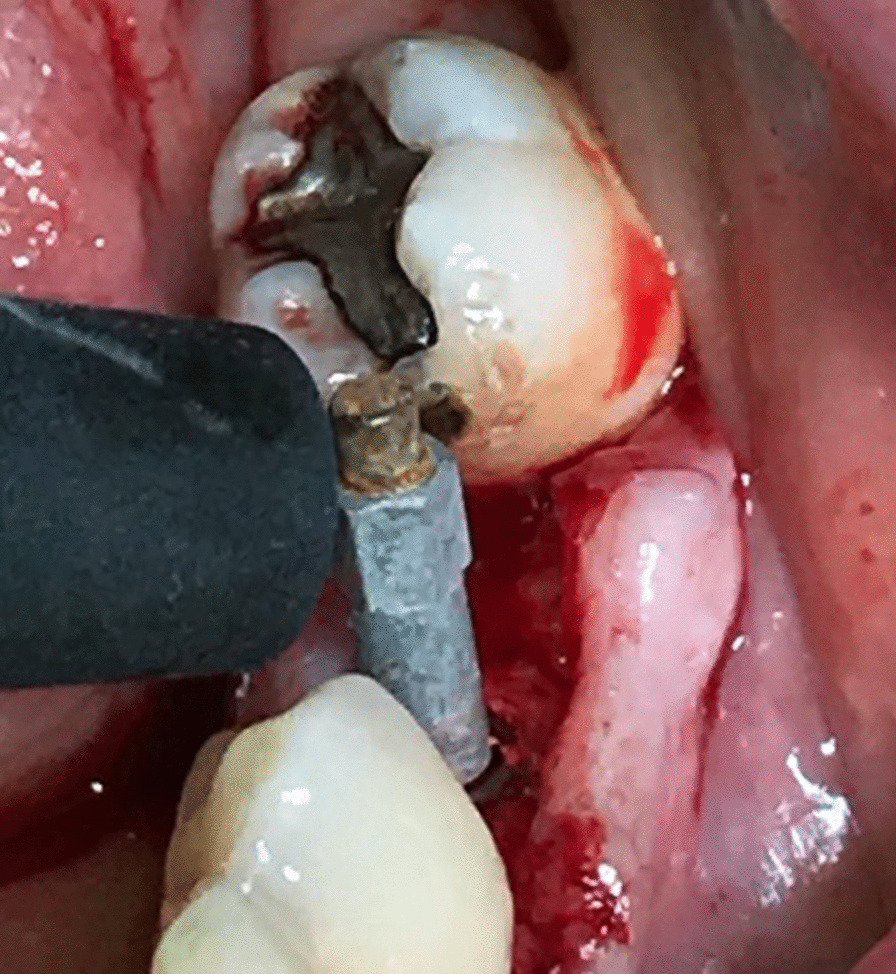
Measuring of the primary stability immediately after implant insertion with the Osstell

The primary outcome measures were implant failure and the implant stability. Implant was judge as failed in case of loss of osseointegration for any reason. The implant stability was measured at baseline (primary implant stability) and 6 weeks after implant placement (secondary stability), by using the Periotest (Medizintechnik Gulden, Modautal, Germany) and the Osstell ISQ module (W&H, Bürmoos, Austria). The Periotest utililizes an electrically driven tapping head, which percusses the implant and the obtained Periotest Value (PTV) indicates implant stability based on the parameters of repercussion (Fig. 1). The Osstell (ISQ) device measures by a resonance frequency analysis while using a special transducer. The device generates oscillation in the transducer via the piezoelectric effect, thus the index is given by the interactions of the implant and the quiver (Fig. 2). After gaining all the data we compared the primary and secondary stability values and registered the differences.

Secondary outcome measures were the following: periimplant probing depth evaluated three months after the definitive prosthesis delivery. Six points around the implants were measured (three measuring points on the buccal side and three on the oral side).

### Statistical analysis

A computer generated randomization scheme was used to allocate patients in three different groups (SA—20 patients, NH—20 patients, SLA—20 patients). Randomization codes were secured in an opaque envelope. An external assessor, not previously involved in the study, prepared all the envelopes.

Data were presented as mean standard deviation and median. A Shapiro–Wilk test was used for testing the normality of the data. The presence of a significant difference between the examined groups (implant stability) was revealed with the Mann–Whitney and Kruskal–Wallis test. Fischer Exact Probability Test or Chi-Square test were performed to assess unbalance between groups and to evaluate dichotomous outcomes. All statistical comparisons were two-tailed and conducted at the 0.05 level of significance. The implant was used as the statistical unit of analysis.

## Results

Of the original 60 enrolled patients seven patients were dropped out. In the SA group four patients were dropped out due to did not come at the planned visits for reasons not related to the present research. Three patients were dropped out in the SLA group due to did not attend the follow up visits for reasons not related to the present research. No patient was dropped out in the NH group. Finally, a total of 75 implants (SA 16, NH 39, SLA 20) were inserted into various positions (6 front, 26 premolar, and 43 molar) in 53 patients (35 women, mean age 44 years and 18 men, mean age 53 years). Ten percent of participants were light smokers (less than 10 cigarettes per day).

In the SA group 6 3.5 mm diameter implants, 8 4.0 mm diameter implants and 2 4.5 diameter implants were inserted. The implant length was 7 mm in 2 cases, 8.5 mm in 4 cases, 10 mm in 6 cases and 11.5 mm in 4 cases. Implants were placed in various positions according to patients’ individual needs (2 front, 6 premolar and 8 molar).

In the NH group 17 3.5 mm diameter implants, 20 4.0 mm diameter implants and 1 4.5 diameter implants were inserted. The implant length was 8.5 mm in 7 cases, 10 mm in 25 cases and 11.5 mm in 6 cases. Implants were placed in various positions according to patients’ individual needs (2 front, 12 premolar and 25 molar).

In the SLA group 10 3.3 mm diameter implants and 10 4.1 mm diameter implants were inserted. The implant length was 8 mm in 3 cases and 10 mm in 17 cases. Implants were placed in various positions according to patients’ individual needs (2 front, 8 premolar and 10 molar). (Table 1).

**Table 1:** Baseline patients' and implants' characteristics

	SA (n = 16)	NH (n = 39)	SLA (n = 20)	*P* value
Smoking patients	1	3	0	0
Maxillary implants	4	9	14	0.002
Mandible implants	12	30	6	
Frontal position	2	2	2	0.73
Premolar area	6	12	8	
Molar area	8	25	10	
3.5/3.3 mm diameter^a^	6	17	10	0.49
4.0/4.1 mm diameter	8	20	10	
4.5 mm diameter	2	1	0	
7 mm length^a^	2	0	0	0.23
8.5/8 mm length	4	7	3	
10 mm length	6	25	17	
11.5 mm length	4	6	0	

^a^Osstem and Straumann respectively

According to the baseline characteristics of the patients and the implants only in case of the implant location in the jaws a significant difference is shown. As a background the patients’ individual needs for dental implant rehabilitation could stand. Since the difference between the primary and secondary stability was measured in this study this value is not influences the results.

### Implant success

However, the primary stability values were suitable (PTV = − 4; ISQ = 67) one NH implant failed spontaneously due to improper surgical technique at the fourth week after implant placement. No implants failed in the other groups. The difference was not statistically different (*p* = 0.99).

### Implant stability

The mean Periotest and Osstell ISQ data of SA, NH and SLA surface-modified implants were reported in the Table 2 (implant placement, baseline) and 3 (prothesis delivery). (Tables 2, 3).

**Table 2:** Mean primary stability immediately after implant placement

Primary stability	PTV (mean ± SD)	ISQ (mean ± SD)
SLA	− 4.75 (± 0.967; median = − 5)	65.95 (± 9.897; median = 68.5)
SA	− 5.23 (± 1.166; median = − 5)	55.69 (± 15.782; median = 48)
NH	− 4.49 (± 1.802; median = − 5)	59.11 (± 19.523; median = 65)

PTV, periotest value; ISQ, implant stability quotient; SD, standard deviation

**Table 3:** Mean secondary stability immediately at the definitive prosthesis delivery

Secondary stability	PTV (mean ± SD)	ISQ (mean ± SD)
SLA	− 5.35 (± 0.745; median = − 5)	67.85 (± 9.906; median = 69.50)
SA	− 5.38 (± 0.957; median = − 5)	63.44 (± 16.789; median = 65)
NH	− 5.10 (± 1.410; median = − 5)	64.10 (± 19.793; median = 66)

PTV, periotest value; ISQ, implant stability quotient; SD, standard deviation

Primary and secondary stability values were compared in each group. Both ISQ values were significantly increased in every groups. However, in case of the PTV values only in the SLA group was a significant change. In the NH and SA groups the results were approaching towards to significance (Tables 4, 5, 6).

**Table 4:** Difference of secondary and primary stability values in SA group

SA	PTV (mean ± SD)	ISQ (mean ± SD)
Primary stability	− 5.23 (± 1.166; median = -5)	57.56 (± 16.240; median = 54,50)
Secondary stability	− 5.38 (± 0.957; median = − 5)	63.44 (± 16.789; median = 65)
Difference	1.13 (± 2.13)	5.88 (± 7.42)
*P* value	0.408	0.009

**Table 5:** Difference of secondary and primary stability values in NH group

NH	PTV (mean ± SD)	ISQ (mean ± SD)
Primary stability	− 4.59 (± 1.802; median = − 5)	58.08 (± 19.526; median = 65)
Secondary stability	− 5.10 (± 1.410; median = − 5)	64.10 (± 19.793; median = 66)
Difference	0.76 (± 1.89)	6.03 (± 17.93)
*P* value	0.045	0.001

**Table 6:** Difference of secondary and primary stability values in SLA group

SLA	PTV (mean ± SD)	ISQ (mean ± SD)
Primary stability	− 4.75 (± 0.967; median = -5)	65.95 (± 9.897; median = 68.50)
Secondary stability	− 5.35 (± 0.745; median = − 5)	67.85 (± 9.906; median = 69.50)
Difference	0.6 (± 0.94)	1.9 (± 3.96)
*P* value	0.002	0.001

Analyzing the three implant surfaces for difference in primary and secondary stability values we found no significant results with either measuring method (ISQ—*p* = 0,338; Periotest—*p* = 0,946). (Table 7).

**Table 7:** Difference between primary and secondary stability in the three examined groups

	PTV (mean ± SD)			ISQ (mean ± SD)		
	SA	NH	SLA	SA	NH	SLA
Difference	1.13(± 2.13)	0.76(± 1.89)	0.6 (± 0.94)	5.88(± 7.42)	6.03(± 17.93)	1.9 (± 3.96)
*P* value	0.946			0.338		

Although the differences between primary and secondary implant stability was higher in the NH group with the ISQ measuring method and in the SA group with the PTV measuring method, there were no significant differences between the three study groups.

### Clinical measurements

Periimplant probing depth yielded favorable outcomes in each implant. No pockets > 5 mm were detected; soft tissue conditions were optimal around every inserted implant. In general, periimplant probing depth was smaller on the buccal surfaces. Higher than average probing depths were detected in two aspects of the oral surface. The highest average value was registered at the the disto-oral surface (0.86). The lowest average probing depth was recorded on the mid-buccal surface (0.63). (Table 8).

**Table 8:** Measurements of the gingival sulcus depth, three months after the implant placement

Periimplant probing depth	Buccal 1 average value	Buccal 2 average value	Buccal 3 average value	Oral 1 average value	Oral 2 average value	Oral 3 average value
3 Months after implant placement	0,69 (± 0.53, median = 1)	0,63 (± 0.598, median = 1)	0,77 (± 0.598, median = 1)	0,71 (± 0.825, median = 1)	0,71 (± 0.667, median = 1)	0,86 (± 0.55, median = 1)

## Discussion

The present randomized trial was aimed to investigate whether the implant surface characteristics influence the osseointegration process up to six weeks healing. The result of the present study showed a significant difference of the implant stability from the baseline to 6 weeks after insertion. There was significant difference in each study groups between the changes of primary and secondary stability values. The values increased after six weeks. However, no differences were found between the tested group, even if a trend of a better result could be refered to the NH implants. In a systematic review Huthayfa and colleagues after investigate 1.137 study, reported that in comparison hydrophilic surface implants to sandblasted, acid-etched surface implants there was no significant effect on improving implant stability [3].

Based on the literature, moderately rough surface could increase the clinical outcome after dental implant placement by facilitating osseointegration [10, 27]. Although the NH and SA surfaces showed a higher roughness (Ra 2–3 μm) compared to the SLA surface, the results of the present research were similar. It could be concluded that all the three groups could be safely used in case of early loading protocol, when the recommended primary implant stability could be achieved during insertion.

In the present research, in all the tested implants primary implant stability statistically increased. The increase of the implant stability was the lowest in case of SLA group and the highest in case of NH group.

The main limitations of the present research are the sort follow-up period and the small sample size, due to a sample size calculation was not performed. Further research should be conducted to better evaluate the osseointegration process at its most critical period. Usually, the value of the primary stability of the inserted implants decreases after two weeks and it approaches its original value after fourth to sixth weeks [6]. At this point, the secondary stability emerges. Presumably, the physiological processes are positively influenced by the different modified surfaces during the first two weeks after dental implant insertion. In a randomized control trial, it was resulted that in case of new hydrophilic surface, the primary stability values significantly increased after two weeks of implant insertion. [24]

In the present study the primary stability was the highest in case of SLA group. The primary stability is not influenced by the implant surface. Possible explanations were related to the implant macro design, the drilling protocol and the bone characteristics. In the present study there was no significant difference between the secondary stability results. The implants were inserted into different quality of bone (D1–D3). The results show that after six weeks of healing good secondary stability could be achieved, allowing for safe early loading protocol with all of these type of surface modifications and in case of D3 bone quality as well. Further research should be conducted in D4 bone quality to evaluate the clinical outcomes even in poor bone quality.

After three months of prothesis delivery good soft-tissue integration and stably of the attached mucosa was recorded. The rates of the periimplant probing depths were higher on the oral surfaces. This can be explained by the fact that the oral surfaces of the teeth are more difficult to brush and clean. This observation reflects how important the frequent recalls and the proper motivation are to achieve long-term success and to prevent the formation of periimplantitis.

## Conclusion

With the limitation of the present research, all the tested implants showed improved stability six weeks after implant placement. A trend of higher result was found for the NH group. Further studies with longer follow-up and sample size are needed to confirm this preliminary results.

## Data Availability

The datasets used and/or analysed during the current study are available from the corresponding author (Kinga Körmöczi, kormoczi.kinga@dent.semmelweis-univ.hu) on reasonable request.

## Abbrevitations

BIC: Bone-implant contact
HA: Hydroxyapatite
ISQ: Implant stability quotient
NH: Nano-hydroxyapaptite
PTV: Periotest value
SA: Sandblasted and acid ached
SD: Standard deviation
SLA: Sand-blasted large grit acid-etched
